# Supplementary figures and images for: RNA-seq: impact of RNA degradation on transcript quantification
Source: BMC Biol. 2014 May 30;12:42. doi: 10.1186/1741-7007-12-42 (PMC4071332; doi:10.1186/1741-7007-12-42)

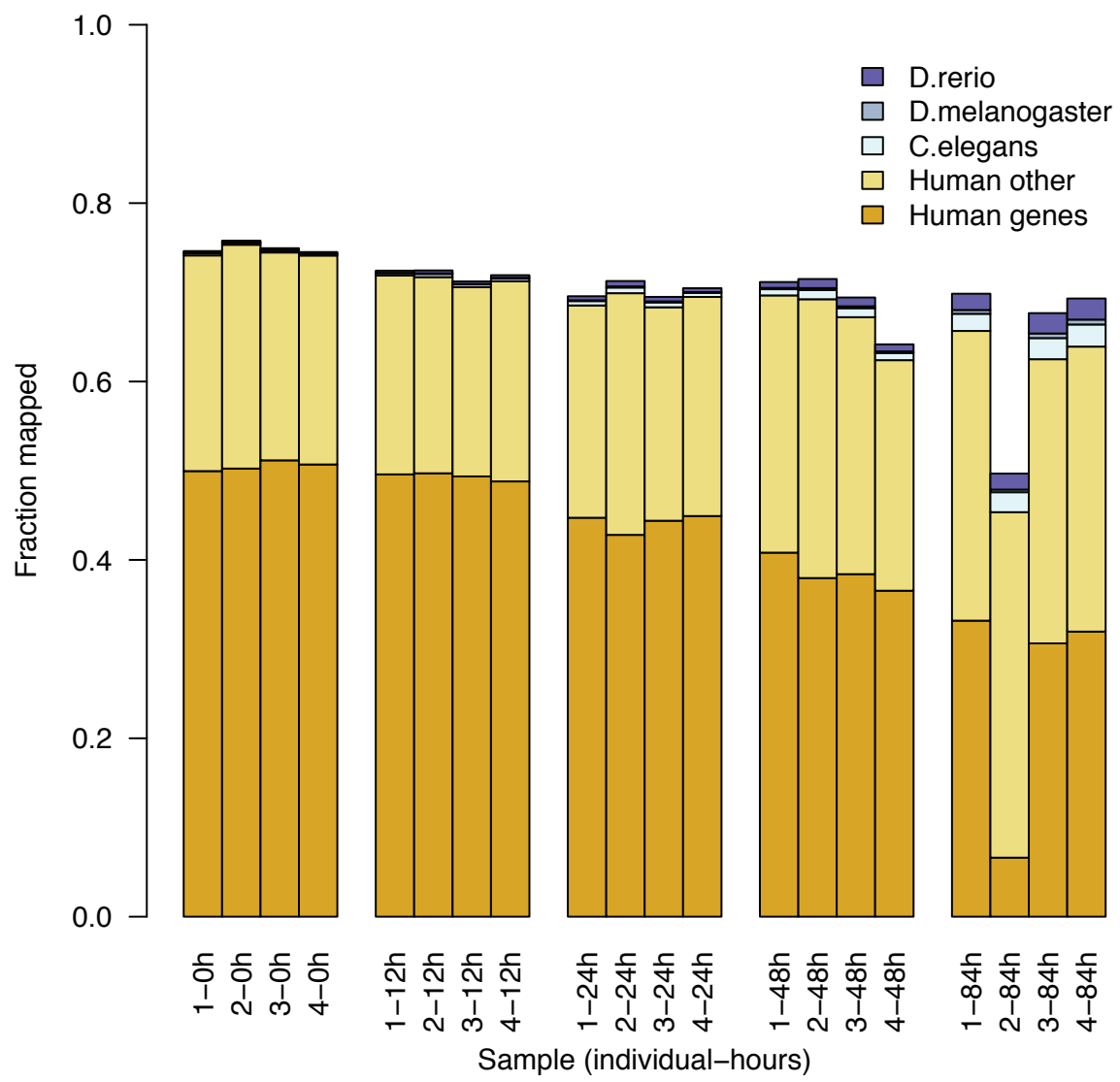

Supplement: Additional file 2: Figure S1 — Fraction of reads mapped from generated libraries. All samples were randomly subset to the same depth prior to mapping. [file 1741-7007-12-42-S2.pdf]

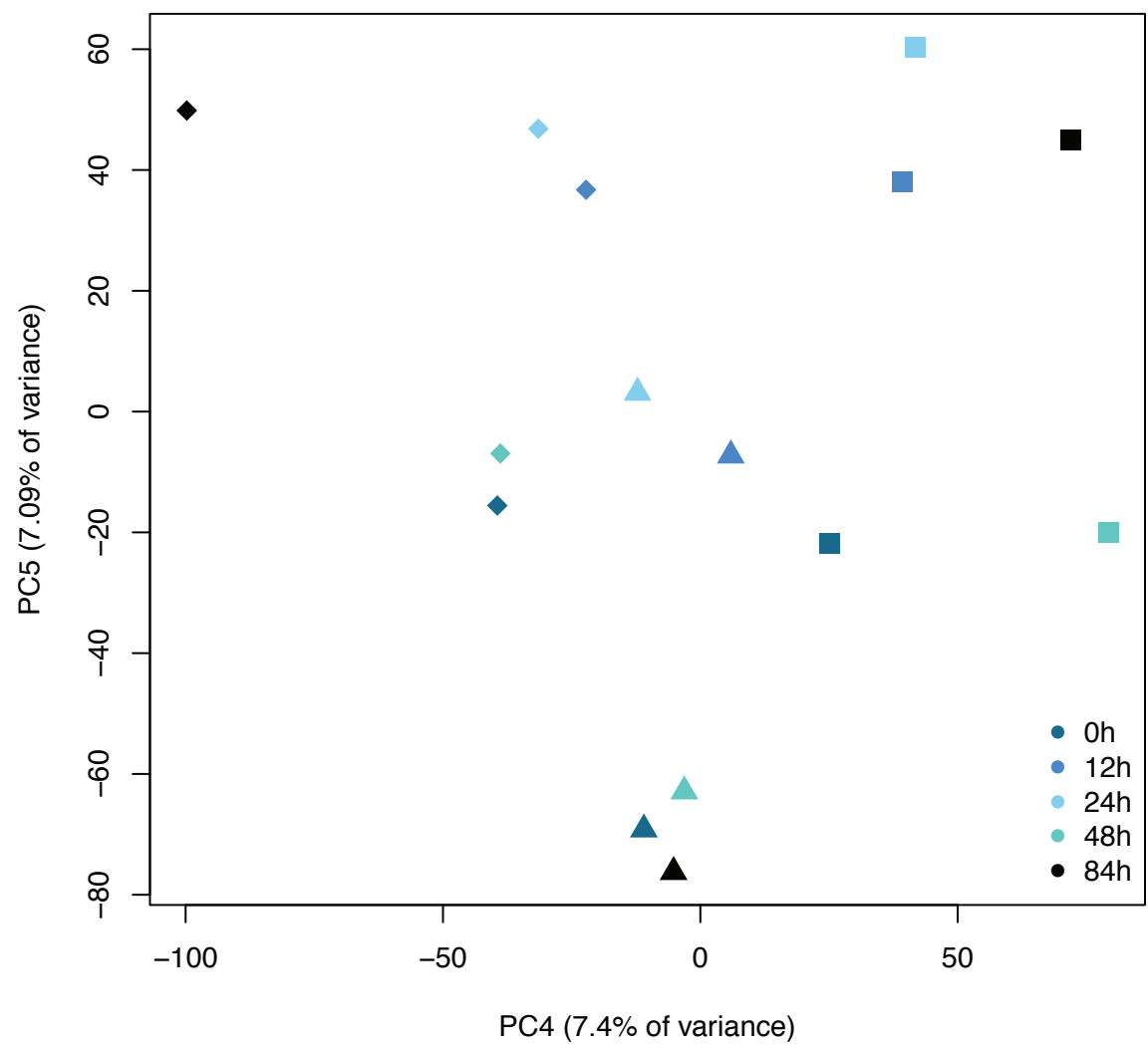

Supplement: Additional file 4: Figure S2 — PCA plot of principal components 4 and 5, the only components significantly associated with inter-individual variation in the data. Different colors identify different time-points, while each shape indicates a particular individual in the data set. [file 1741-7007-12-42-S4.pdf]

A.

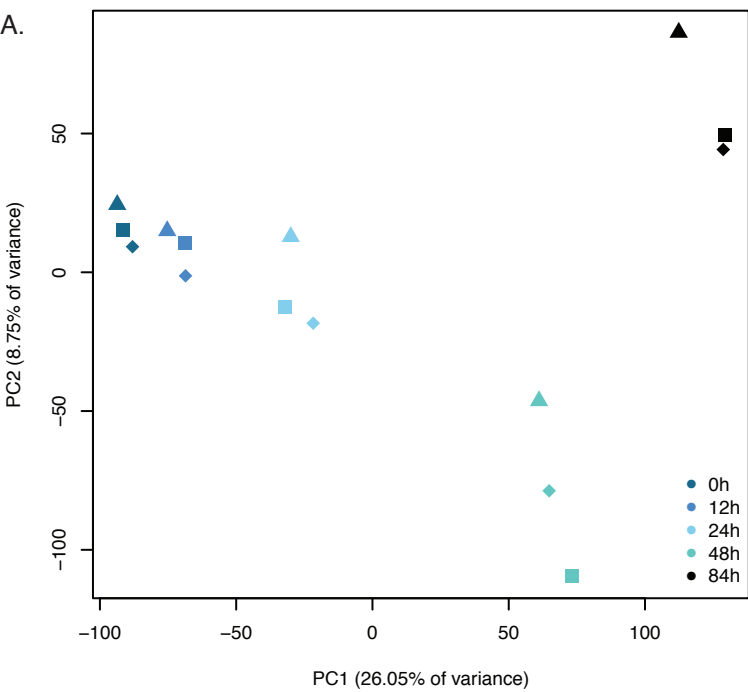

B.

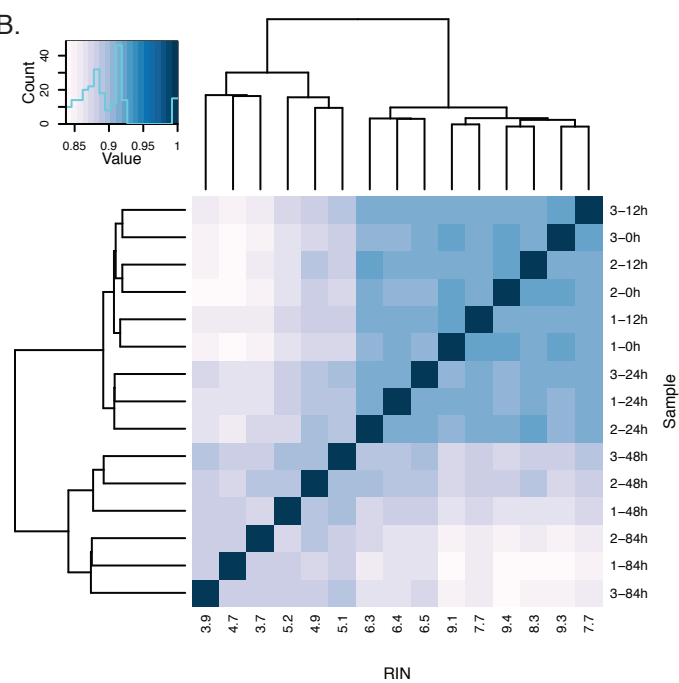

Supplement: Additional file 5: Figure S3 — A) PCA plot of the 15 samples included in the study based on data from 27,856 genes with at least one mapped read to the 1,000-most 3′ base pairs in a single individual. Different colors identify different time-points, while each shape indicates a particular individual in the data set. B) Spearman correlation plot of the 15 samples in the study, using only data trimmed to the 1,000-most 3′ bp. [file 1741-7007-12-42-S5.pdf]

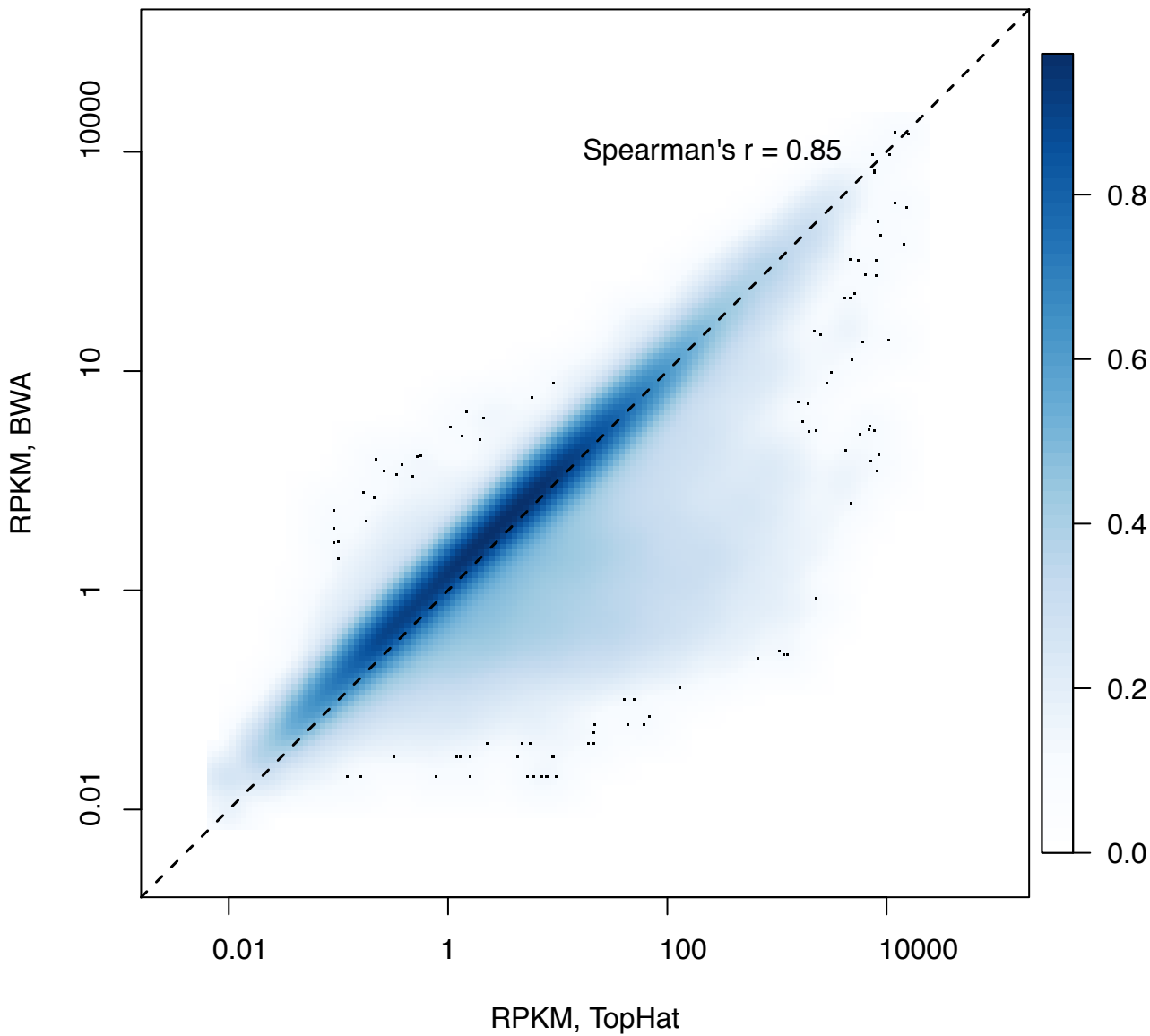

Supplement: Additional file 6: Figure S4 — Density plot of RPKM estimates per gene after mapping with BWA and TopHat. Only genes with an RPKM > =0.3 after mapping with BWA are shown. [file 1741-7007-12-42-S6.pdf]

A.

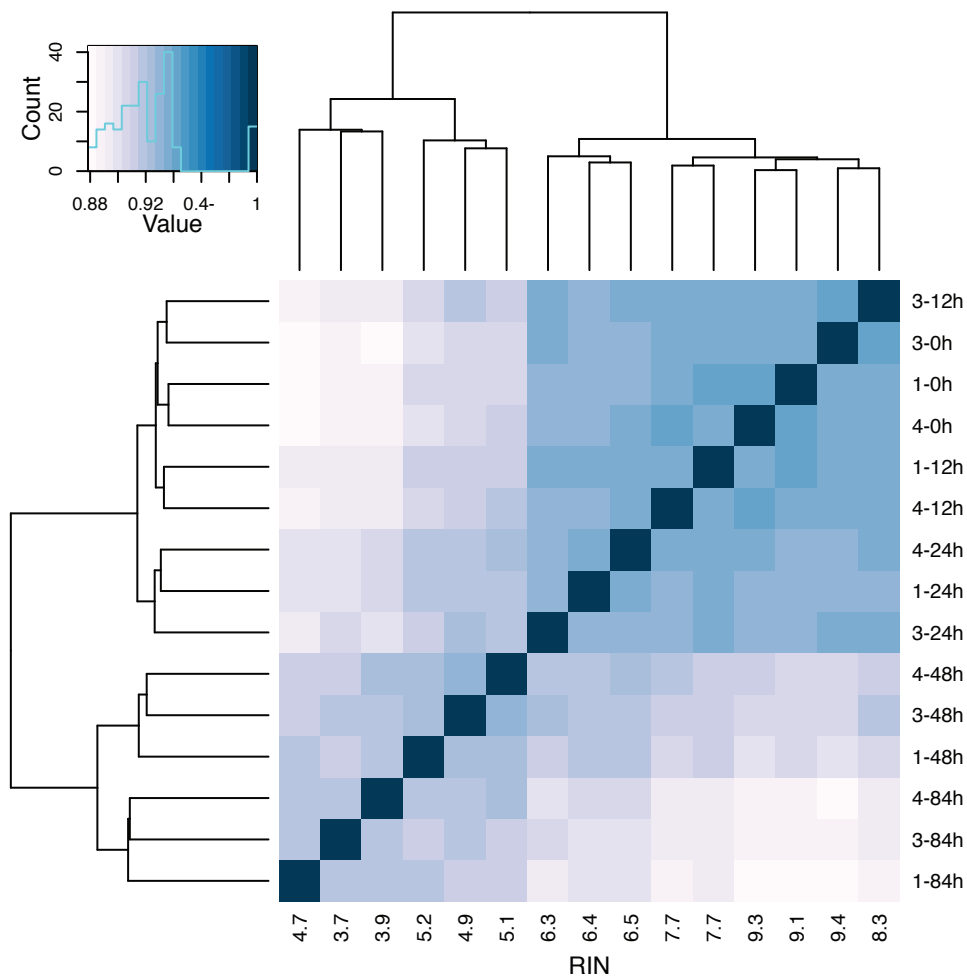

B.

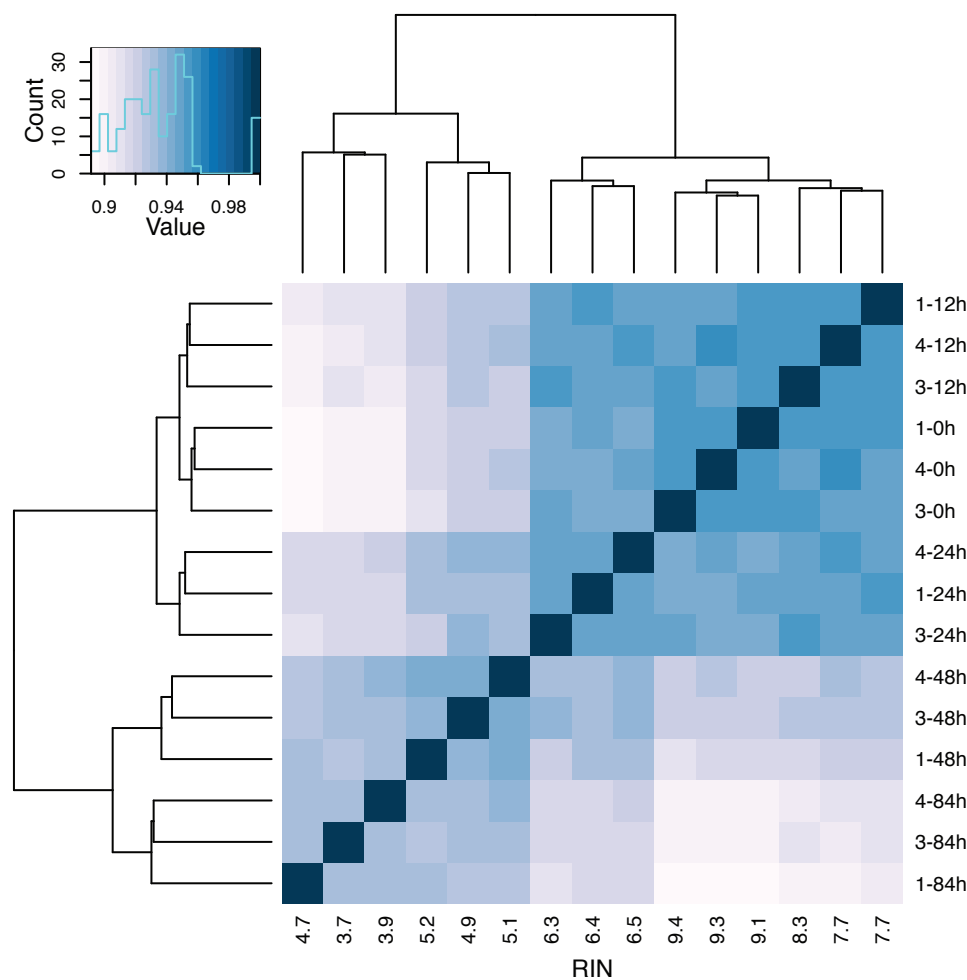

Supplement: Additional file 7: Figure S5 — Spearman correlation plot as in Figure 1 using data mapped by TopHat. A) Correlations across 33,438 genes with at least one instance of one read mapped by TopHat. B) Correlations across 29,156 genes with at least one instance of one read mapped by BWA. [file 1741-7007-12-42-S7.pdf]

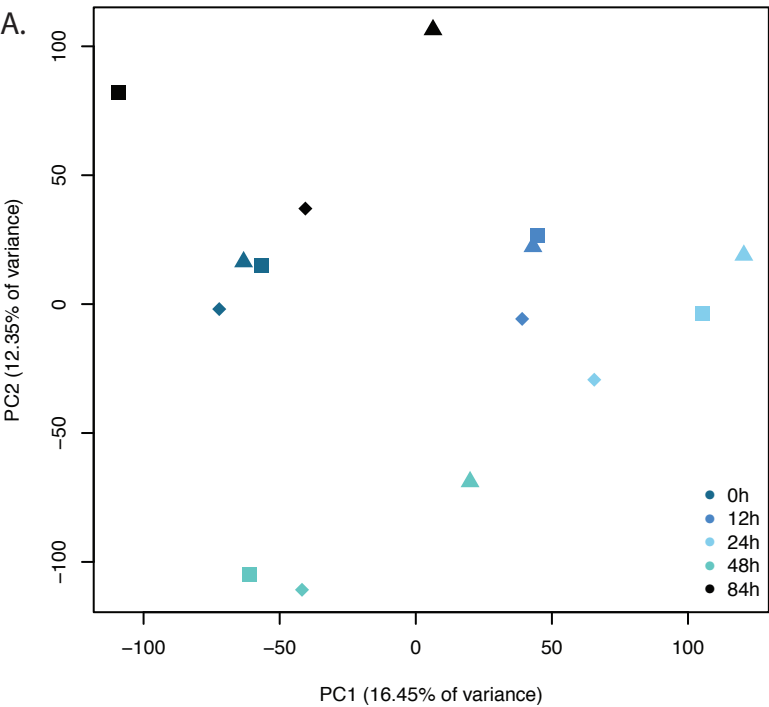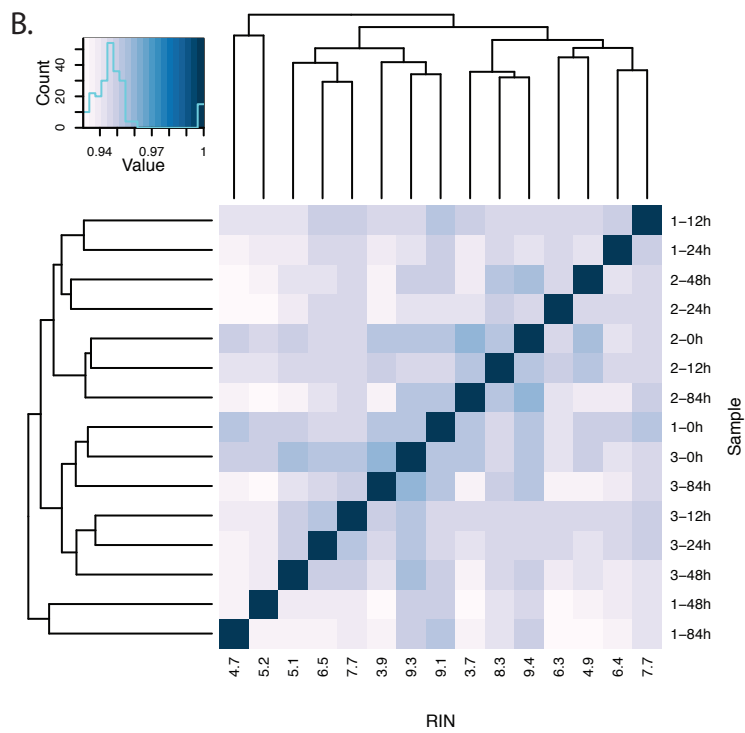

Supplement: Additional file 8: Figure S6 — A) PCA plot of the 15 samples included in the study based on data from 29,156 genes with at least one mapped read in a single individual, after correcting for the effects of RIN on the data. Different colors identify different time-points, while each shape indicates a particular individual in the data set. B) Spearman correlation plot of the 15 samples in the study, after correcting for the effects of RIN on the data. [file 1741-7007-12-42-S8.pdf]

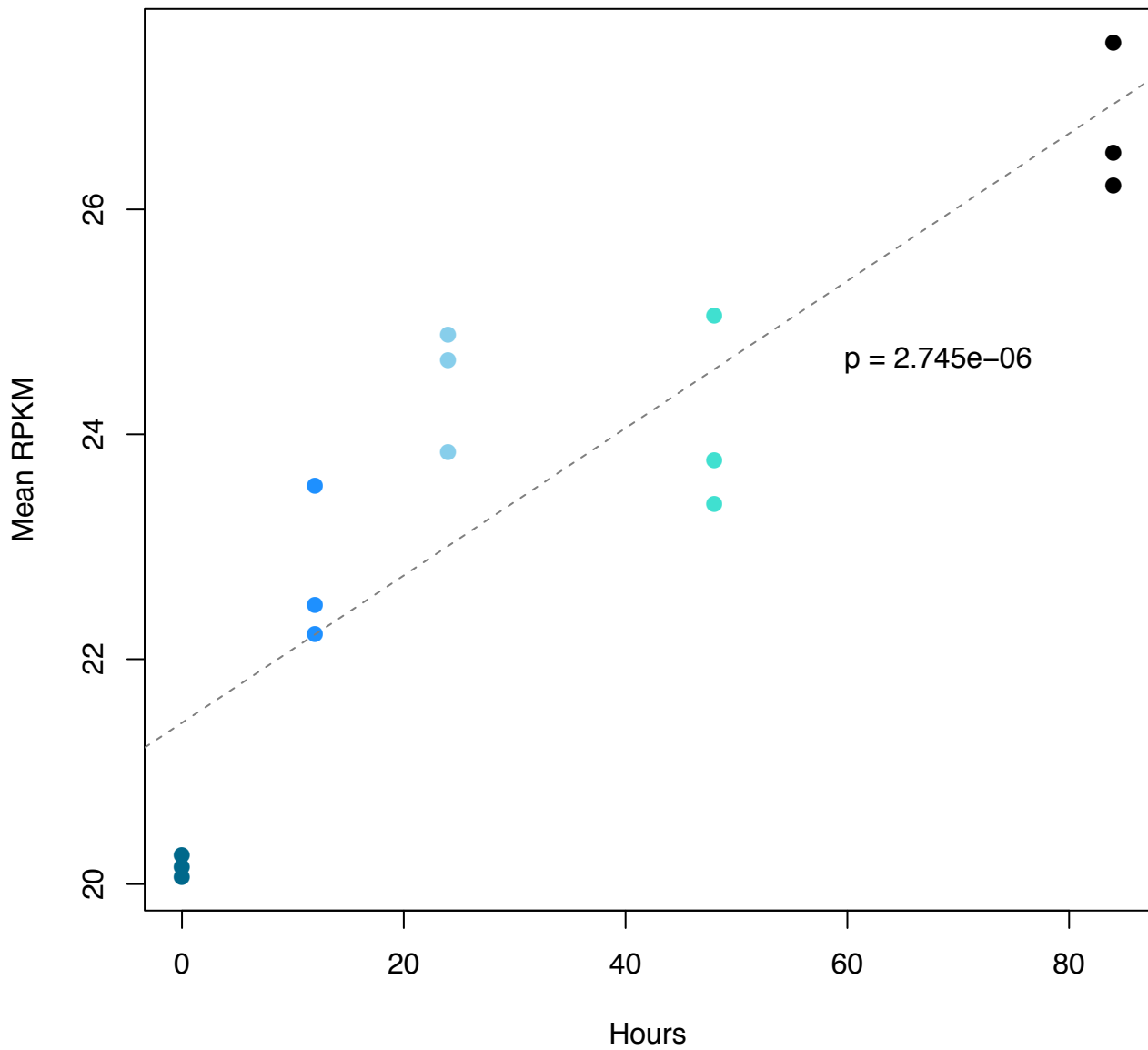

Supplement: Additional file 9: Figure S7 — Mean RPKM as a function of time (h) to sample collection. [file 1741-7007-12-42-S9.pdf]

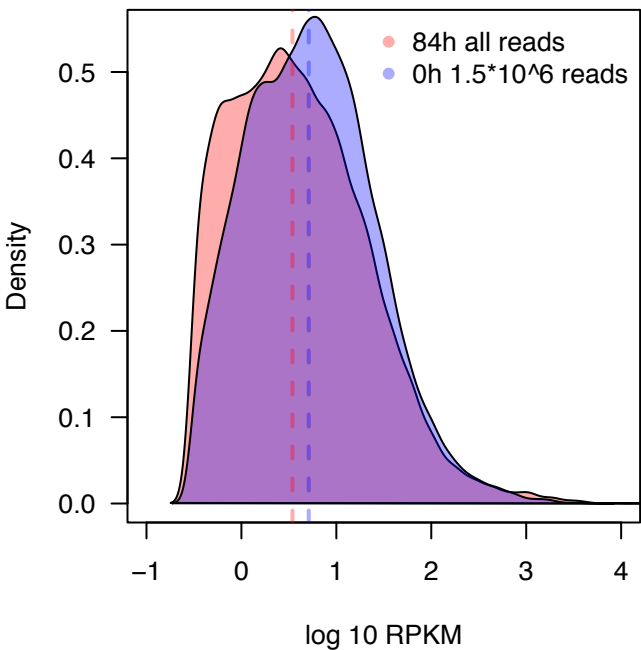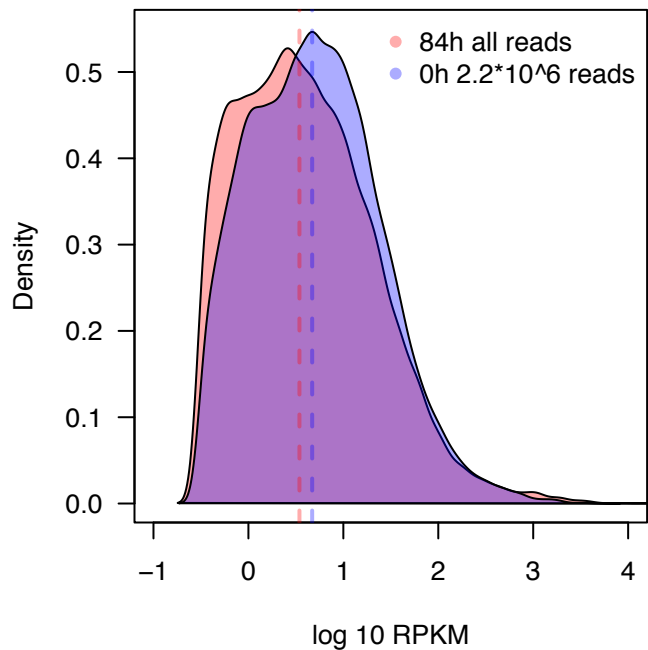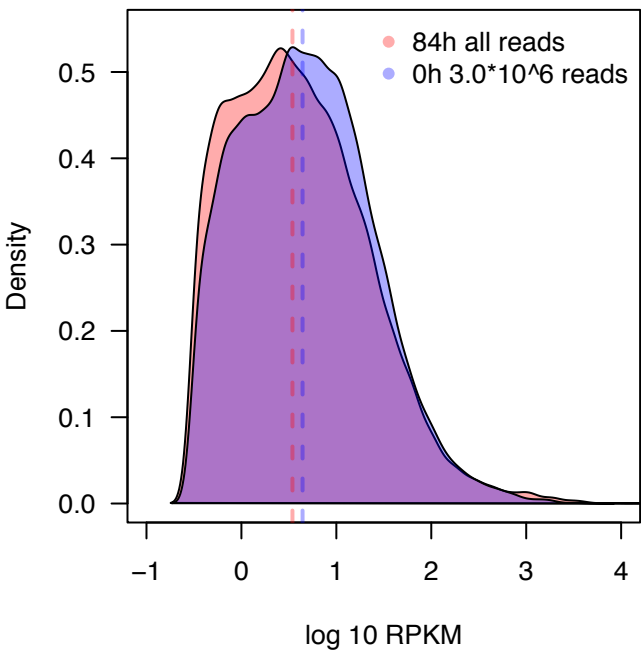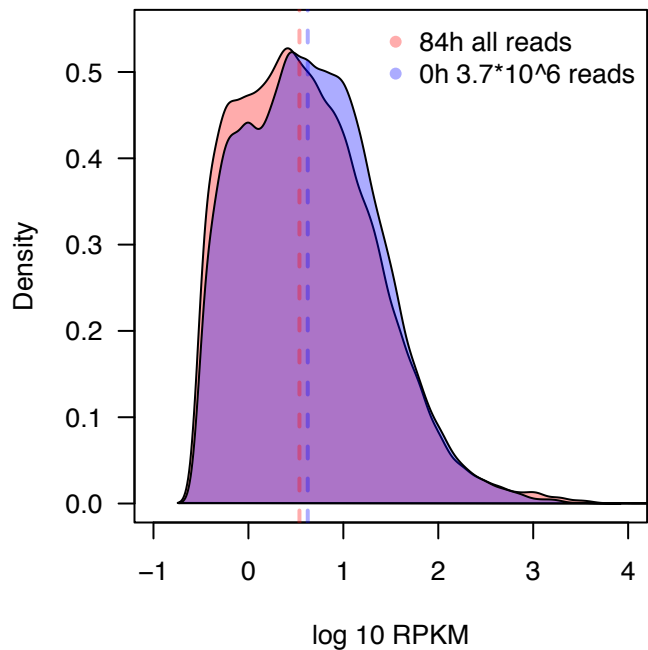

Supplement: Additional file 10: Figure S8 — Effects of sequencing depth on library complexity. Dashed red lines indicate median RPKM in each subset. (A to D) Density plots of RPKM values in the 0-hour data when subsampled to indicated depths. For comparison, the observed distribution of RPKM values in the 84-hour data is plotted in each figure in blue. [file 1741-7007-12-42-S10.pdf]
